# Severe Hyponatremia as an Early Manifestation of Peritransplant Abscess After Living Donor Kidney Transplantation: A Case Report

**DOI:** 10.7759/cureus.116505

**Published:** 2026-09-18

**Authors:** Aaron Huang, Rachael Molina, Navdeep Kaur, Dawn Francisquini, Adam M Kressel

**Affiliations:** 1 Department of Urology, State University of New York at Stony Brook, Stony Brook, USA; 2 Renaissance School of Medicine, State University of New York at Stony Brook, Stony Brook, USA; 3 Department of Nephrology, State University of New York at Stony Brook, Stony Brook, USA

**Keywords:** acute kidney injury, escherichia coli, hyponatremia, kidney transplantation, peritransplant abscess, postoperative complications

## Abstract

Electrolyte abnormalities are common after kidney transplantation, but severe hyponatremia as an early sign of occult peritransplant infection is rare. A 68-year-old woman underwent living unrelated kidney transplantation and was discharged on postoperative day 4 with a drain in place. On postoperative day 8, she developed acute kidney injury and profound hyponatremia, with a serum sodium level of 109 mmol/L, despite only mild fatigue and no fever, abdominal pain, allograft tenderness, or wound symptoms. Sodium improved with hypertonic saline, nutritional support, and fluid restriction. On postoperative day 13, she developed fever; computed tomography revealed a 16-cm peritransplant abscess, and drainage grew *Escherichia coli*. This case highlights severe unexplained hyponatremia as a potential early manifestation of occult infectious or surgical complications after kidney transplantation.

## Introduction

Hyponatremia, defined as a serum sodium level <135 mmol/L, is a common electrolyte disturbance in hospitalized patients and is associated with increased morbidity, prolonged hospitalization, and adverse outcomes. Symptoms vary by acuity and severity, ranging from nausea, fatigue, and malaise to seizures, coma, and death [1]. However, even profound hyponatremia may be minimally symptomatic.

Kidney transplant recipients are especially vulnerable to electrolyte disturbances due to perioperative volume shifts, evolving allograft function, immunosuppression, antimicrobial prophylaxis, and comorbid disease. Hyponatremia is typically attributed to hypovolemia, impaired free-water excretion, medication effects, endocrine dysfunction, or reduced solute intake [2]. In the setting of infection, hyponatremia is often secondary to inappropriate arginine vasopressin release, dysregulation of the renin-angiotensin-aldosterone system, and subsequent decrease in free water excretion [3]. Occult post-transplant infection as a presenting cause is less commonly recognized.

We report a case of profound, minimally symptomatic hyponatremia early after living donor kidney transplantation in a patient later found to have a large peritransplant abscess requiring percutaneous drainage.

## Case presentation

A 68-year-old woman with stage V chronic kidney disease secondary to biopsy-proven focal segmental glomerulosclerosis underwent living unrelated kidney transplantation. Her medical history included hypertension, hyperlipidemia, hypothyroidism, nonobstructive coronary artery disease, osteoporosis treated with denosumab, gastroesophageal reflux disease, previously treated *Helicobacter pylori* gastritis, small intestinal bacterial overgrowth, bullous pemphigoid, herpes simplex virus type II infection, and anemia. Before transplantation, she was maintained on low-dose prednisone and had recently transitioned from cyclosporine to mycophenolate mofetil.

The transplant procedure was completed without immediate surgical complication. Induction immunosuppression included alemtuzumab, methylprednisolone, and mycophenolate mofetil. The donor kidney had two arteries: the superior renal artery was anastomosed to the recipient left external iliac artery, and the inferior renal artery to the recipient left inferior epigastric artery. Cold ischemia time was less than three hours, and total anastomosis time was 30 minutes. She received four units of packed red blood cells and one unit of fresh frozen plasma perioperatively. She resumed oral intake on postoperative day 1, regained bowel function on postoperative day 3, and was discharged on postoperative day 4 with a surgical drain in place. Drain output was less than 100 mL in the 24 hours before discharge. Maintenance immunosuppression included tacrolimus, prednisone, and mycophenolate mofetil.

On postoperative day 6, she reported one episode of emesis with mild nausea during transplant clinic evaluation. Telephone follow-up on postoperative day 7 revealed symptom improvement. On postoperative day 8, she returned with increased serosanguineous drain output of 365 mL over 24 hours. Fluid analysis was consistent with serum. Laboratory testing demonstrated acute kidney injury, with serum creatinine increased to 2.55 mg/dL from 1.44 mg/dL at discharge, and severe hyponatremia, with a serum sodium level of 110 mmol/L. She was referred urgently to the emergency department.

Repeat testing confirmed a serum creatinine of 2.47 mg/dL and a sodium of 109 mmol/L. Despite these abnormalities, she reported only fatigue and denied fever, abdominal pain, nausea, incisional pain, incisional drainage, allograft tenderness, or other localizing symptoms. Transplant kidney ultrasonography showed no hydronephrosis, normal resistive indices, and a small peritransplant fluid collection.

She was admitted with presumed hypovolemic hyponatremia attributed to decreased oral intake. Admission urine studies showed urine sodium <20 mmol/L, urine potassium 18 mmol/L, urine chloride <25 mmol/L, and urine osmolality 297 mOsm/kg. Calculated serum osmolality was 240.46 mOsm/kg, with serum sodium 109 mmol/L, glucose 115 mg/dL, and blood urea nitrogen 45 mg/dL. Thyroid-stimulating hormone was 2.399 μIU/mL, and cortisol was 14.9 μg/dL. Hypertonic saline was initiated and later transitioned to nutritional support with fluid restriction. Serum sodium corrected to 134 mmol/L over five days.

On postoperative day 13, she developed a fever to 38.6 °C. Infectious evaluation included blood and urine cultures, chest radiography, bilateral lower-extremity venous duplex ultrasonography, and abdominopelvic imaging. Computed tomography demonstrated a 16-cm peritransplant collection inferior and anterior to the renal allograft, separate from the existing drain, concerning for abscess (Figures 1-2).

**Figure 1:**
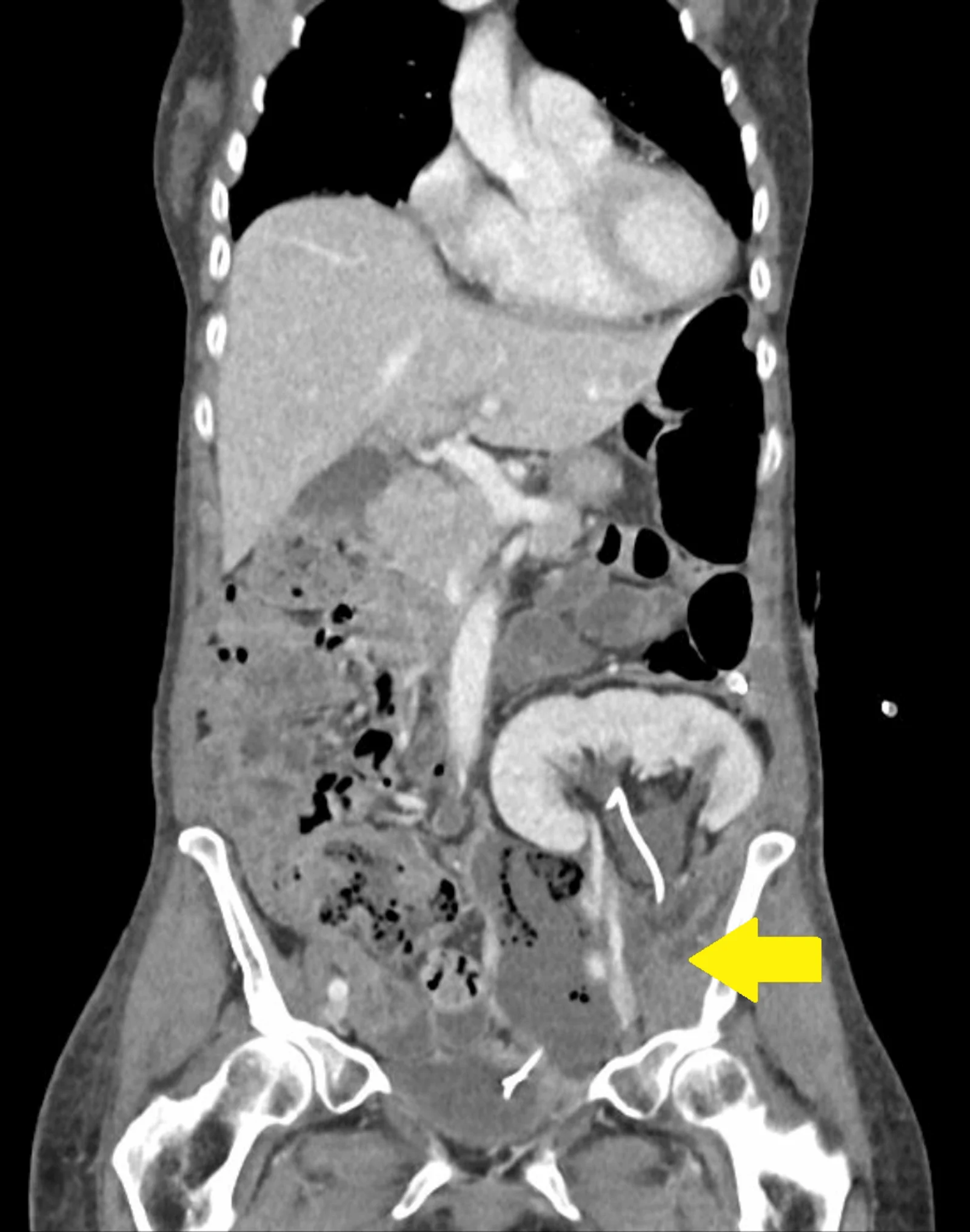
Coronal CT scan demonstrating a large peritransplant abscess. A stent is seen in place. The kidney is noted to be rotated due to the fluid collection. Yellow arrows denote a perirenal abscess collection in both the coronal and sagittal planes.

**Figure 2:**
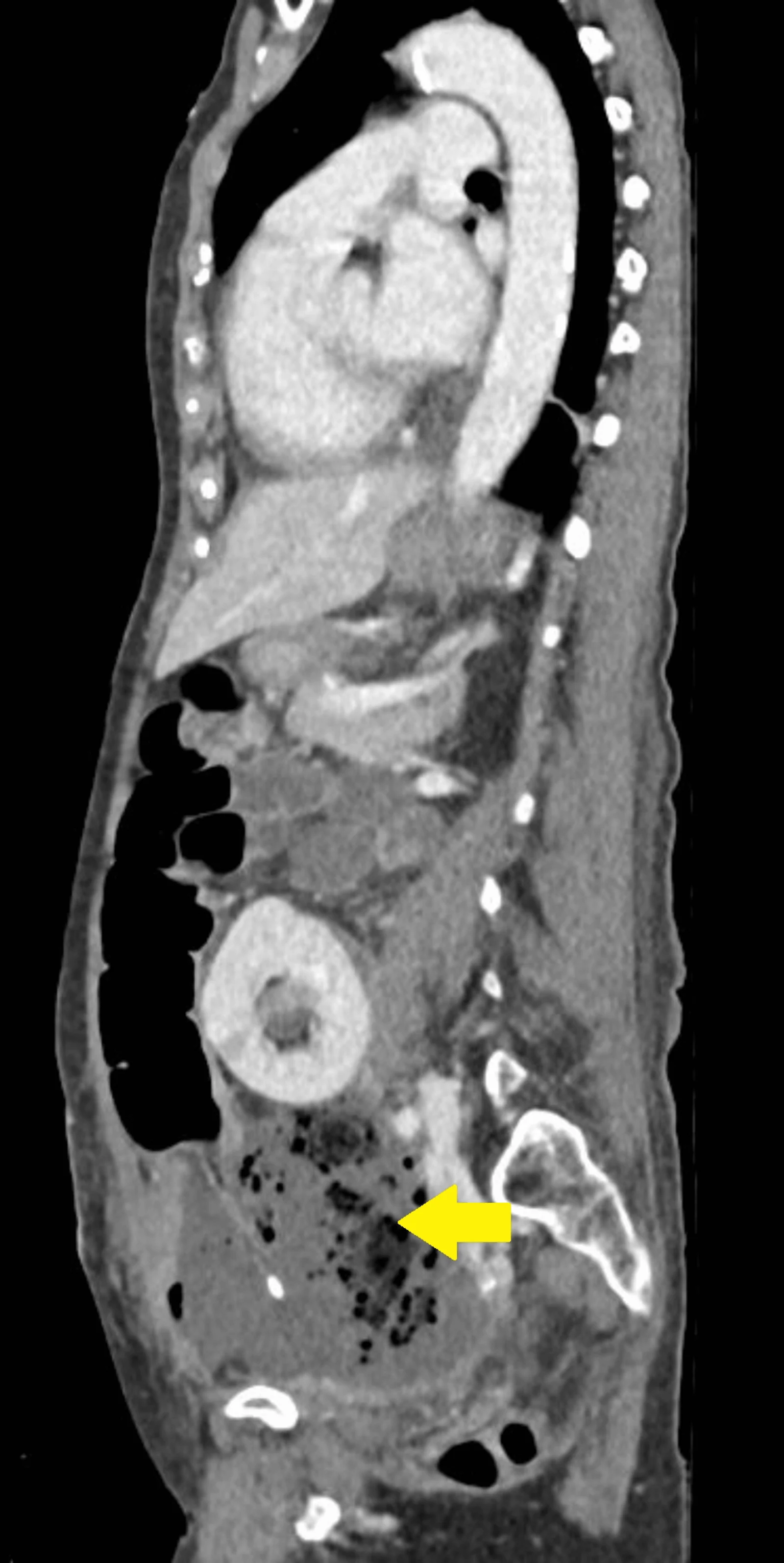
Sagittal CT scan demonstrating a large peritransplant abscess. A stent is seen in place. The kidney is noted to be rotated due to the fluid collection. Yellow arrows denote a perirenal abscess collection in both the coronal and sagittal planes.

Interventional radiology performed percutaneous drainage, yielding cloudy pink fluid for culture.

After drainage, fever resolved and serum sodium normalized to 136 mmol/L the following morning. Fluid culture grew *Escherichia coli*, and antimicrobial therapy was tailored to susceptibilities. She was discharged on postoperative day 16, hospital day 8, on oral cefpodoxime in stable condition. She was followed closely in the transplant clinic and by interventional radiology with serial imaging. Both drains were subsequently removed, the collection resolved, and serum creatinine returned to baseline.

## Discussion

This case illustrates severe hyponatremia as an early and clinically prominent manifestation of occult peritransplant abscess after kidney transplantation. Although electrolyte abnormalities are common in this population, the degree of hyponatremia was profound and initially occurred without fever, abdominal pain, allograft tenderness, incisional findings, or other localizing signs of infection.

Peritransplant and renal allograft abscesses are uncommon but clinically important complications [4]. They typically present with fever, abdominal or graft-site pain, leukocytosis, graft dysfunction, or constitutional symptoms [5,6]. In this case, however, the initial presentation was dominated by laboratory abnormalities - severe hyponatremia, acute kidney injury, and increased drain output - rather than overt infection. The discovery of a large culture-positive abscess, followed by clinical improvement and sodium normalization after drainage and antimicrobial therapy, supports infection as an important contributor.

The hyponatremia was likely multifactorial. Initial urine studies, recent nausea, and reduced oral intake were consistent with hypovolemia. However, occult infection may also promote hyponatremia through systemic inflammation, nonosmotic arginine vasopressin release, reduced effective arterial circulating volume, nausea, and impaired free-water excretion. In transplant recipients, these mechanisms may be further compounded by perioperative fluid shifts, evolving allograft function, immunosuppression, antimicrobial exposure, and reduced solute intake [7].

This case highlights the limitations of relying solely on symptoms or initial ultrasonography in immunosuppressed patients. Transplant recipients may have attenuated inflammatory responses, and infections may initially lack fever or localizing findings. Although ultrasound identified only a small peritransplant collection, computed tomography demonstrated a much larger collection separate from the surgical drain, enabling targeted source control.

Severe hyponatremia early after kidney transplantation should not be attributed solely to oral intake, perioperative volume shifts, or medication effects without continued reassessment [8]. Severe or persistent biochemical abnormalities, particularly when accompanied by graft dysfunction or abnormal drain output, should prompt consideration of surgical or infectious complications. Cross-sectional imaging should be considered when laboratory abnormalities are disproportionate to the apparent clinical presentation or when ultrasound does not fully explain the clinical course.

Although this single case cannot establish causality, the temporal relationship between abscess drainage, clinical improvement, and sodium normalization suggests that occult infection was a meaningful contributor to the patient’s hyponatremia.

## Conclusions

Peritransplant abscess is an uncommon but serious complication after kidney transplantation. Severe, unexplained hyponatremia - even when minimally symptomatic - may be an early manifestation of occult infection. When accompanied by acute kidney injury, increased drain output, or evolving systemic findings, clinicians should consider early cross-sectional imaging to evaluate for peritransplant collections requiring source control.
